# Clinical characteristics of hospitalized patients with *Nocardia* genus detection by metagenomic next generation sequencing in a tertiary hospital from southern China

**DOI:** 10.1186/s12879-023-08615-z

**Published:** 2023-11-08

**Authors:** Yingjian Liang, Minmin Lin, Lidi Qiu, Meizhu Chen, Cuiyan Tan, Changli Tu, Xiaobin Zheng, Jing Liu

**Affiliations:** 1 Department of Pulmonary and Critical Care Medicine (PCCM), Fifth Affiliated Hospital of Sun Yat-Sen University, 52 East Meihua Rd, Zhuhai City, 519000 Guangdong Province China; 2 Department of Infectious Disease Intensive Care Unit, Fifth Affiliated Hospital of Sun Yat-Sen University, 52 East Meihua Rd, Zhuhai City, 519000 Guangdong Province China; 3 Department of General Intensive Care Unit, Fifth Affiliated Hospital of Sun Yat-Sen University, 52 East Meihua Rd, Zhuhai City, 519000 Guangdong Province China

**Keywords:** Clinical features, *Nocardia*, Metagenomic next generation sequencing, Infection diseases, Bronchoalveolar lavage fluid

## Abstract

**Objective:**

As an opportunistic pathogen, *Nocardia* often occurring in the immunocompromised hosts. As the unspecifc clinical presentation and low identification rate of the culture dependent methods, *Nocardia* infection may be under-diagnosis. Recent study have reported physicians could benefit from metagenomic next-generation sequencing (mNGS) in *Nocardia* diagnosis. Herein, we present patients with a positive detection of *nocardiosis* in mNGS, aiming to provide useful information for an differential diagnosis and patients management.

**Methods:**

A total of 3756 samples detected for mNGS from March 2019 to April 2022 at the Fifth Affifiliated Hospital of Sun Yat-sen University, were screened. Clinical records, laboratory finding, CT images and mNGS results were reviewed for 19 patients who were positive for *Nocardia* genus.

**Results:**

Samples from low respiratory tract obtained by bronchoscope took the major part of the positive (15/19). 12 of 19 cases were diagnosis as Nocardiosis Disease (ND) and over half of the ND individuals (7/12) were geriatric. Nearly all of them (10/12) were immunocompetent and 2 patients in ND group were impressively asymptomatic. Cough was the most common symptom. *Nocardia cyriacigeorgica* (4/12) was more frequently occurring in ND, followed by *Nocardia abscessus* (3/12). There are 3 individuals detected more than one kind of *Nocardia* species (Supplementary table 1). Except one with renal failure and one allergic to sulfamethoxazole, all of them received co-sulfonamide treatment and relieved eventually.

**Conclusion:**

Our study deciphered the clinical features of patients with positive *nocardiosis* detected by mNGS. Greater attention should be paid to the ND that occurred in the immunocompetent host and the geriatric. Due to the difficulties in establishing diagnosis of Nocardiosis disease, mNGS should play a much more essential role for a better assessment in those intractable cases. Co-sulfonamide treatment should still be the first choice of Nocardiosis disease.

**Supplementary Information:**

The online version contains supplementary material available at 10.1186/s12879-023-08615-z.

## Introduction

*Nocardia* species are ubiquitous groups of gram-positive environmental bacteria which were initially reported in 1888 [1, 2]. As a gram-positive bacterium that grows aerobically, it can be found in soil, decomposing vegetation, and other organic matter, as well as in fresh and salt water. *Nocardia* is generally considered as an opportunistic pathogen, with the majority of infections occurring in the immunocompromised hosts, like malignancies, human immunodeficiency virus infection, solid-organ or hematopoietic stem cell transplant and those receiving long-term treatment with steroids or other immunosuppression, but it can cause life-threatening disease in the immunocompetent patients [2, 3]. *Nocardia* is capable of causing a variety of infections, including pulmonary nocardiosis, central nervous system (CNS) infection, cutaneous disease and bloodstream infection (BSI). The most common clinical presentation is pulmonary nocardiosis as inhalation is the primary route of bacterial exposure, and the most frequently attacked sites among extrapulmonary is CNS. *Nocardia* bacteremia is less often encountered and it has often concurrent other infected site, such as respiratory tract and skin [2].

The definitive diagnosis of nocardial disease depends on isolation and culture of *Nocardia* from suspected sites [4]. Conventional culture methods include the paraffin baiting method and there are several commonly used media like normal microflora paraffin agar, Lowenstein Jensen medium, Ogawa agar, blood agar and modified Thayer Martin medium [5–9]. However, due to the interference and coexistence of other microorganism as well as the complicated experimental procedures, *Nocardia* identification rate in respiratory samples is still extremely low [10]. Moreover, clinical presentation of nocardial disease may be subacute to chronic, and symptoms can include cough, dyspnea, hemoptysis, pyrexia, chest pain, night sweats, weight loss, and progressive fatigue. Regarding chest radiograph, pulmonary nocardiosis can be variable, which displaying focal or multifocal disease with nodular and/or consolidation infifiltrate, cavitary lesions and pleural effusions [4]. As the unspecifc clinical presentation, chest imaging, the inexperience of the clinical laboratory technicians in primary hospitals in China, the particular features of *Nocardia* growth, and the low sensitivity of the culture-based method, *Nocardia* infection maybe not diagnosed in a timely manner [10].

Recently, several studies had found that the state-of-the-art technology, metagenomic next-generation sequencing (mNGS) or next-generation sequencing (NGS), had a satisfactory diagnostic value compared to conventional methods in *nocardiosis* diagnosing and it could assist clinical decision making with minimized turnaround time [11, 12].

However, mNGS positive results sometimes are not taken into consideration of clinical physicians as contamination of clinical specimens and colonization of the respiratory tract may occur [4]. Therefore, a diagnosis of Nocardiosis Disease often depends on experience of physicians, in spite of mNGS can provided reliable reads of *nocardiosis*. Herein, we present patients with a positive detection of *nocardiosis* in mNGS, including final diagnosis as Nocardiosis Disease (ND) and no-Nocardiosis Disease(nND), aiming to provide useful information for an differential diagnosis and patients management.

## Methods

### Ethics statement

A total of 3756 samples detected for mNGS from March 2019 to April 2022 at the Fifth Affifiliated Hospital of Sun Yat-sen University (a tertiary hospital in Zhuhai city in the south of China) were conducted a retrospective review. This retrospective study was approved by the Research Ethics Committee of the Fifth Affiliated Hospital of Sun Yat-sen University (approve number [2022] K82-1). Written informed consent for participation was not required for this study in accordance with the national legislation and the institutional requirements.

### Study design

Demographic data and clinical information on patients with a positive detection of *nocardiosis* in mNGS was collected. Patients were seperated in two groups, ND and nND, according to the final diagnosis of first medical contact or first mNGS results.

### Sample collection, processing and storage

The collection process of bronchoalveolar lavage fluid (BALF)/sputum/cerebrospinal fluid(CSF)/blood/abscess were in line with standard operating procedure and followed the aseptic principle. During the interim period, all the samples kept refrigerated and immediately transferred to -80 °C freezer.

### DNA extraction and mNGS sequencing

Attached to a horizontal platform on a vortex mixer, 1.5 mL microcentrifuge tube with 0.6 mL sample of BALF/sputum/CSF/abscess and 250μL 0.5 mm glass bead were agitated vigorously at 2800–3200 rpm for half an hour. Next, 7.2μL lysozyme was added for wall-breaking reaction. TIANamp Micro DNA Kit (DP316, TIANGEN BIOTECH) was used to extracted DNA following the manufacturer’s operational manual.

3 mL of blood were placed in blood collection tube and stored at room temperature for 3–5 min before plasma separation and centrifuged at 4,000 rpm for 10 min at 4℃ within 8 h of collection. After transferred to new sterile tubes, DNA was extracted from 300 uL of plasma using the TIANamp Micro DNA Kit (DP316, TIANGEN BIOTECH, Beijing, China) according to the manufacturer’s recommendation [13].

DNA libraries were constructed through DNA-fragmentation, end-repair, adapter-ligation and PCR amplification. Agilent 2100 was used for quality control of the DNA libraries. Quality qualified libraries were pooled, DNA Nanoball (DNB) was made and sequenced by BGISEQ-50 /MGISEQ-2000 platform [14].

### Bioinformatic analysis

High-quality sequencing data were generated after removing low-quality reads, according to computational substraction of human host sequences mapped to the human reference genome (hg19) by Burrows-Wheeler Alignment [15]. The remaining data by removal of low-complexity reads were classified by simultaneously aligning to Pathogens metagenomics Database (PMDB), consisting of bacteria, fungi, viruses and parasites. The classification reference databases were downloaded from NCBI (ftp://ftp.ncbi.nlm.nih.gov/genomes/). RefSeq contains 4,945 whole genome sequence of viral taxa, 6,350 bacteral genomes or scaffolds, 1064 fungi related to human infection, and 234 parasites associated with human diseases.

## Result

### Samples collecting

**Fig. 1:**
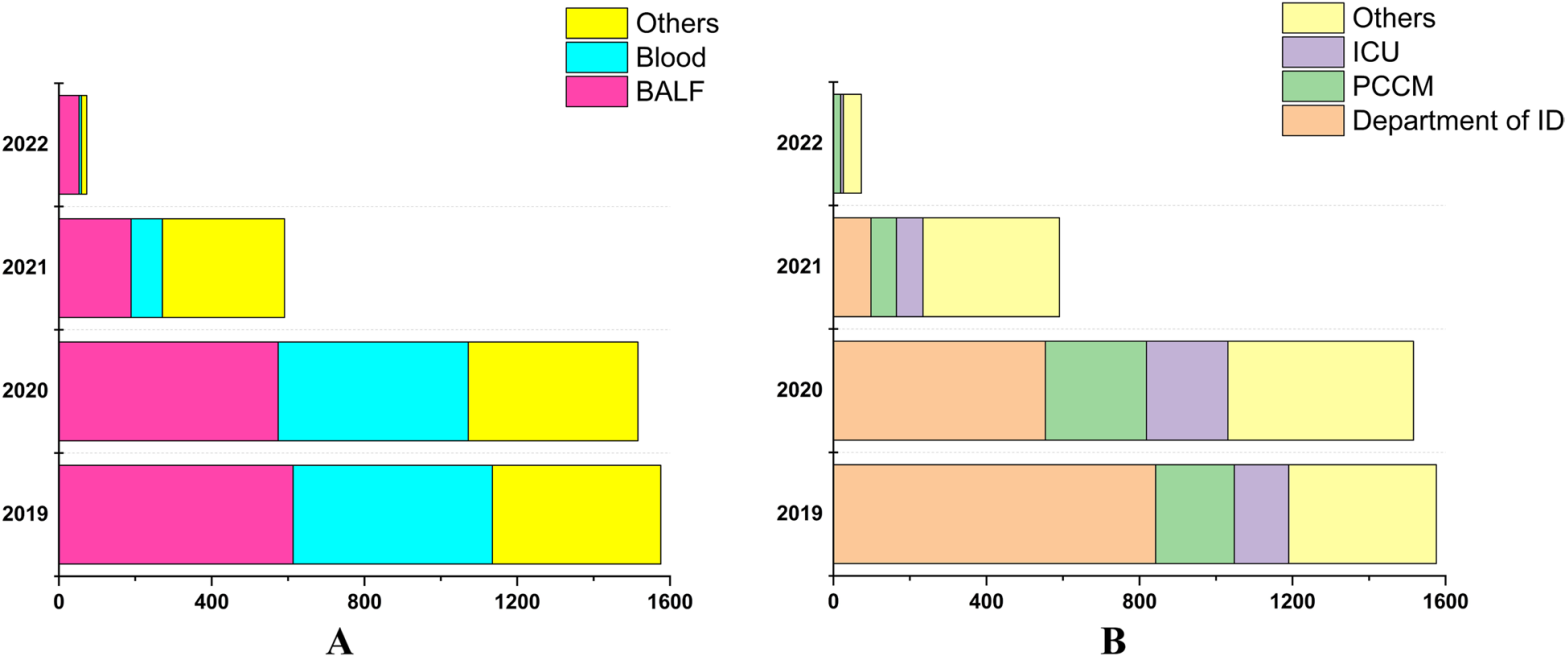
Distribution of samples collecting from March 2019 to April 2022. **A** Seperated sites. BALF, Bronchoalveolar Lavage Fluid. **B** Seperated departments from March 2019 to April 2022. ID, Infectious Diseases, ICU, intensive care unit, PCCM, pulmonary and critical care medicine

**Fig. 2:**
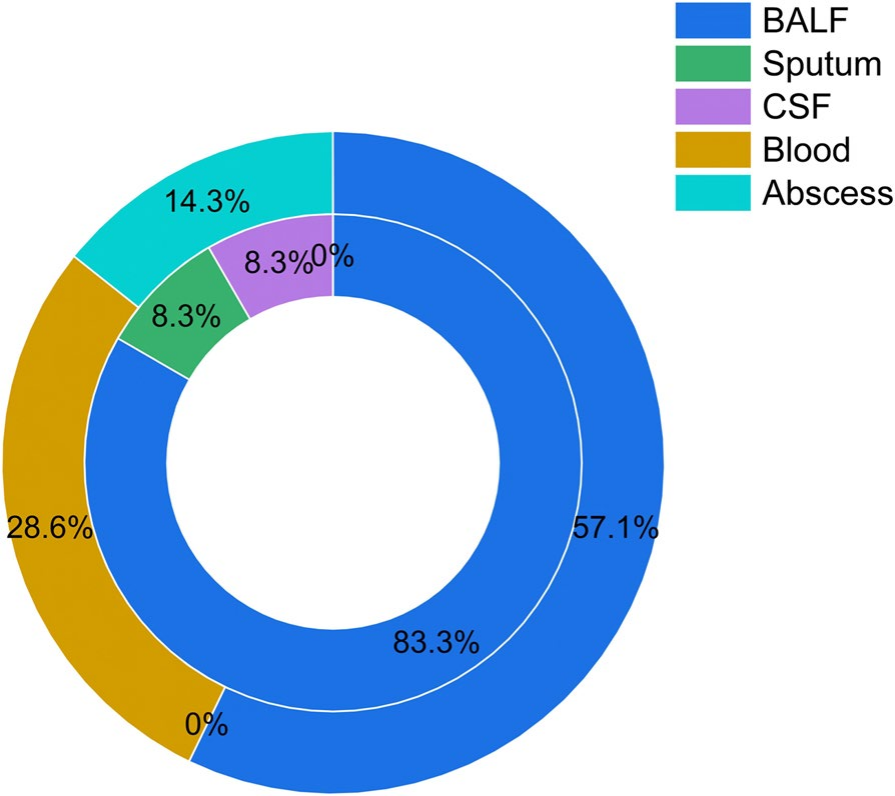
Distribution of seperated sites. Inner Circle respresents Group of ND. Outer Circle respresents Group of nND. BALF, Bronchoalveolar Lavage Fluid. CSF, Cerebrospinal Fluid

BALF as well as blood samples accounted for a large part and department of infectious diseases (ID) as well as pulmonary and critical care medicine (PCCM) performed most of the mNGS testing in our hospital (Fig. 1). Of the 3756 samples assessed, mNGS results from 19 patients were positive for *nocardiosis* genus and distribution as well as baseline characteristics were illustrated as Table 1 and Fig. 2. Samples from low respiratory tract obtained by bronchoscope took the major part of the positive. Over half of positive samples (10/19, 52.63%) as well as the ND patients (7/12, 58.33%) were from 2020.

**Table 1:** Demographics and clinical characteristics of study patients

	**ND, *n* = 12**	**nND, *n* = 7**
**Gender**		
** Male**	7	4
** Female**	5	3
**Age**		
** 31–60**	5	4
** 60 + **	7	3
**Sample Year**		
** 2019**	3	3
** 2020**	7	3
** 2021**	0	1
** 2022**	2	0
**Ward Type**		
** Department of ID**	6	3
** Department of PCCM**	4	1
** ICU**	1	1
** Others**	1	2
** Mean departments experienced before definite diagnosis**	1.5	1.43
**Main Complaint**		
** Cough**	9	3
** Hemoptysis**	2	1
** Pyrexia**	1	4
** Dyspnea**	2	1
** Asymptomatic**	2	1
** Smoking History**	4	0
** Immunocompromised**	3	3
** Solid organ transplantation**	1	0
** Immunosuppressive agents usage**	1	1
** Chemo- or radiation therapy of malignancy**	1	2
**Previous Medical History**		
** Hepatobiliary disease**	7	4
** Urological disease**	6	3
** Bronchiectasia**	3	2
** Diabetes**	0	2
** Hypertension**	2	1
** COPD**	1	0
** Means days between admission and mNGS detected**	6.25	10.29
**Outcomes**		
** Relieved**	10	6
** Against-advice discharged**	2	1

### Demographics characteristics and clinical presentation

All of the patients were over 30-year-old with a average of 59.94 years old. 9/12 of the ND individuals were over 50 years old and 7/12 were geriatric (over 60 years old). Half of the ND were admitted to department of ID and before the definite diagnosis, more than one department was experienced for patients on average. Individuals in the ND group had a higher proportion of smoking. The most prevalent symptom among ND was cough and several patients in ND group were asymptomatic. One of asymptomatic individual (Patient C) visited our facility due to the routine check after chemotherapy of nasopharyngeal carcinoma (NPC) and the other one (Patient I) performed preoperative computerized tomography (CT) scan before the operation of hepatic masses. Only 2 cases were immunocompromised for various reasons. Besides Patient C, Patient A was on long-term use of immunosuppressive medication, including tacrolimus, mycophenolate mofetil and methylprednisolon, after experienced renal transplant surgery. Means days between admission and mNGS detection of ND were shorter than the nND group. Most of ND individuals discharged according to medical advice uneventfully.

### Laboratory finding and chest CT imaging features

Mainly laboratory tests as well as chest CT features were exhibited as the Table 2. There was a higher mean value of alanine aminotransferase (ALT), lactate dehydrogenase (LDH) and serum Creatinine (Scr) in nND. No one was positive in G or GM tests in the ND group while parts of them were positive in tuberculous infection of T cell spot test (T-SPOT) (3/12, 25%). Only 2 of them were confirmed as ND via culture methods. Regarding ND group, radiological examination of the lungs with CT demonstrates various lesions, involved in single or multiple lobes and nearly half of the ND patients presented bilateral multi-lobe involvements.

**Table 2:** Main Laboratory Results and chest CT features on Admission

	**ND**	**nND**
**Laboratory Tests (Mean ± SD)**		
**Blood routine test**		
White blood cells (× 10^9/L)	9.02 ± 5.58	10.88 ± 3.88
Neutrophil count	7.12 ± 6.01	8.89 ± 3.05
Lymphocyte count	1.24 ± 0.72	1.23 ± 0.88
**Blood chemistry**		
Alanine aminotransferase, ALT (U/L)	19.36 ± 12.67	38.14 ± 41.47
Lactate dehydrogenase, LDH (U/L)	222.38 ± 118.70	413.67 ± 345.73
Albumin (g/L)	36.93 ± 7.89	29.63 ± 8.00
Serum Creatinine, Scr (μmol/L)	103.31 ± 91.06	135 ± 174.65
**Infection**		
Procalcitonin (ng/ml)	2.41 ± 2.79	1.33 ± 2.53
C-reactive protein (mg/L)	33.59 ± 71.13	/^a^
GM test (number of positive)	0	1
G test (number of positive)	0	1
T-spot	3	1
Culture (number of positive for *Norcardia*)	2	1
**Chest CT Scan**		^b^
**Involvement Of Lobes**		
**Only Left Upper**	2	0
**Only Left Lower**	1	0
**Only Right Upper**	2	0
**Only Right Middle**	0	0
**Only Right Lower**	2	0
**Bilateral Multi-lobe**	5	6
**Major CT findings**		
**Only Nodular**	3	0
**Only Cavitary lesions**	2	0
**Only Bronchiectasis**	3	1
**Only Consolidation Infiltrate**	1	2
**Pleural effusions**	2	0
**Multiple displays**	3	3

^a^Only one patient tested for CRP

^b^One patient did not perfromed chest CT

### Characteristics of mNGS results

Among the 12 patients in ND group, mNGS detected 8 *Nocardia* species in total and 2 of them was detected *Nocardia* as sole pathogen. Two mNGS tests can only identified *Nocardia* genus with low reads. There were 7 patients detected with merely single *Nocardia* species and there were 2 *Nocardia* species isolated in the remaining 3 patients. *Nocardia cyriacigeorgica* (4 patients) was more frequently occurring in ND, followed by *Nocardia abscessus* (3 patients). Besides *Nocardia* genus, 18 other bacteria genus, 2 fungi genus and 3 viruses were detected in ND group. *Actinomyces* was the most common bacteria together with *Nocardia* and the other microorganisms were sporadic. Regarding nND group, mNGS detected 5 *Nocardia* species with 11 other bacteria genus, 4 fungi genus, 4 viruses and 1 *Mycobacterium tuberculosis* complex in total. *Nocardia farcinica* was most common in nND (3 patients) and almost all the patients were detected 1 *Nocardia* species (6 patients). (Tables 3 and 4, Supplementary Table 1 and Supplementary Table 2).

**Table 3:** Number of Genus detected by mNGS

	**ND**		**nND**	
	**Genus**	**Num**	**Genus**	**Num**
**Bacteria**	*Nocardia* as sole agent	2	*Nocardia* as sole agent	2
	*Nocardia*	12	*Nocardia*	7
	*Actinomyces*	4	*Rothia*	1
	*Haemophilus*	2	*Streptococcus*	2
	*Streptococcus*	2	*Corynebacterium*	2
	*Porphyromonas*	2	*Veillonella*	1
	*Pseudomonas*	1	*Haemophilus*	2
	*Megasphaera*	1	*Prevotella*	1
	*Klebsiella*	1	*Neisseria*	1
	*Prevotella*	1	*Pseudomonas*	1
	*Campylobacter*	1	*Capnocytophaga*	1
	*Veillonella*	1	*Acinetobacter*	1
	*Anaeroglobus*	1	*Porphyromonas*	1
	*Stenotrophomonas*	1		
	*Olsenella*	1		
	*Mycobacterium*	1		
	*Streptococcus*	1		
	*Parvimonas*	1		
	*Bacteroides*	1		
	*Fretibacterium*	1		
**Fungus**	*Nakaseomyces*	1	*Aspergillus*	1
	*Candida*	1	*Penicillium*	1
			*Pneumocystis*	1
			*Candida*	1
**Virus**	*Human gammaherpesvirus 4(EBV)*	2	*Human betaherpesvirus 5(CMV)*	2
	*Human betaherpesvirus 5(CMV)*	2	*Humangammaher* pesvirus4(EBV)	1
	*Human betaherpesvirus 6B*	1	*Human alphaherpesvirus 3(Varicella zoster virus)(VZV)*	1
			*Human alphaherpes virus 1(HSV1)*	1
**Others**			*Mycobacterium tuberculosis complex*	1

**Table 4:** List of *Nocardia* spieces detected by mNGS

**ND**^a^			**nND**		
**Spieces**		**Num. of Reads**	**Spieces**		**Num. of Reads**
*Nocardia cyriacigeorgica*	Patient L	9078	*Nocardia farcinica*	Patient N	28
	Patient H	6		Patient O	7
	Patient E	340		Patient Q	861
	Patient C	6	*Nocardia nova*	Patient P	3
*Nocardia abscessus*	Patient K	796		Patient S	36
	Patient A	30	*Nocardia niwae*	Patient M	79
	Patient D	5	*Nocardia cyriacigeorgica*	Patient R	26
*Nocardia niwae*	Patient B	51	*Nocardia araoensis*	Patient M	18
*Nocardia araoensis*	Patient B	7			
*Nocardia farcinica*	Patient I	1986			
*Nocardia carnea*	Patient E	4			
*Nocardia terpenica*	Patient F	167			
*Nocardia asiatica*	Patient A	2320			

^a^Two mNGS results only can identified nocardia genus with low reads

### Diagnosis, treatment and outcome

**Fig. 3:**
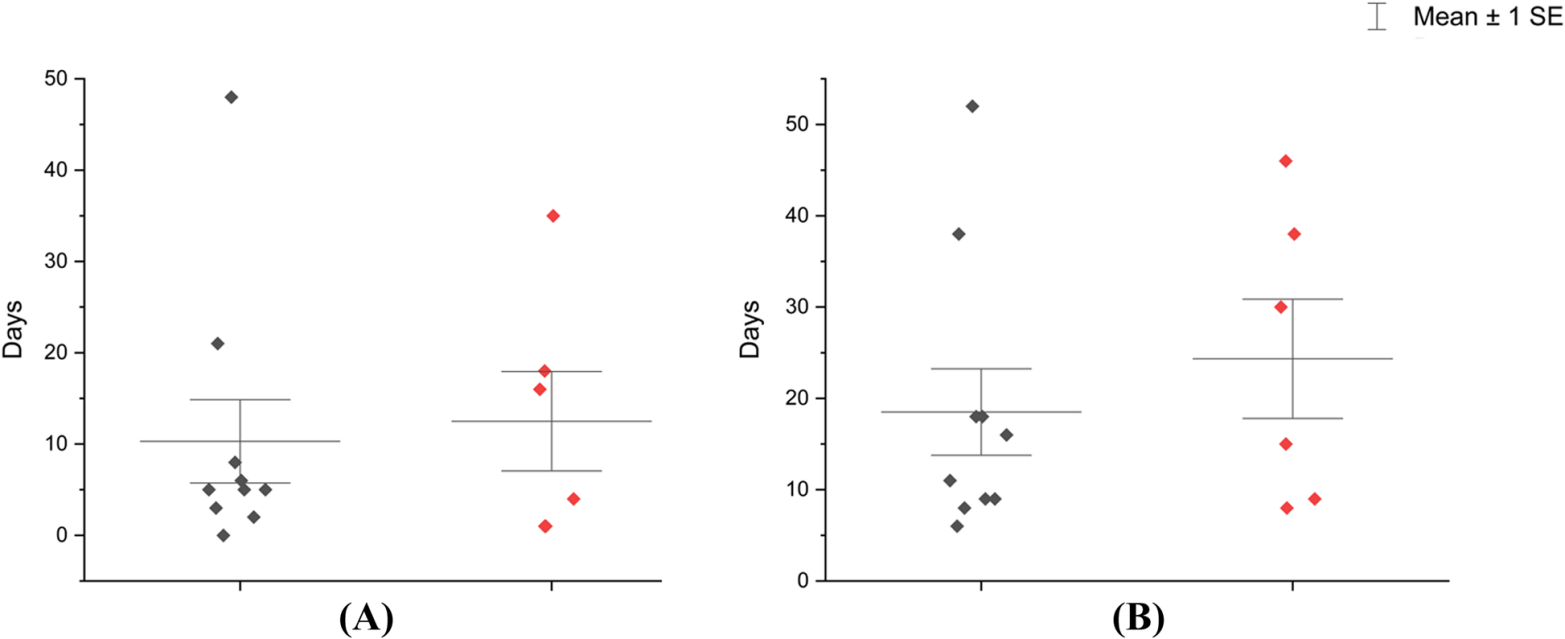
**A** Duration between mNGS results and discharge. **B** Total length of hospital days. Black represents Diagnosed as ND group. Red represent nND group. Patients discharge against medical advice were excluded

Of the 12 patients among ND, all were suffered pulmonary disease and one case was considered as disseminated Nocardiosis Disease (cerebral nocardiosis accompanied with pulmonary nocardiosis). Notably, none of them were firstly considered as ND at admission. Before the diagnosis of ND was established, half of them (6/12) received at least one kind of antibiotic regimens. Two of them were performed anti-tuberculosis treatment (Patient D and Patient G) as well as two of them received anti-fungal therapy (Patient A and Patient C). One third of them initially received empirical antibiotic treatment of consisted of piperacillin-tazobactam (Patient A, C, H and L) or ceftriaxone (Patient B, F, G and J). After ND established, 9 patients (Patient B, C, D, F, G, I, J, K, L) received monotherapy or combination therapy contained of sulfamethoxazole. Patient E was allergic to sulfamethoxazole and thus changed to minocycline. Patient H discharged against-advice earlier than mNGS report came back. Patient A received antibiotic treatment regimens of linezolid and faropenem because of ND combined with extended-spectrum beta-lactamase producing strain of *klebsiella pneumoniae* pneumonia according to the advice of experienced infectious diseases specialist (Table 5). Among the nND group, 2 were diagnosed as tuberculosis (TB) and 1 was pulmonary aspergillosis. Most of them were considered to received antibiotic therapy as infectious diseases, excluded Patient P, who was due to fever after chemotherapy (Table 6). Most of the ND patients could leave hospital within 10 days after mNGS results feedback while the nND was uncertain (Fig. 3).

**Table 5:** Treatment of the patients diagnosed as ND

	**Main diagnosis at admission**	**Diagnosis after mNGS**	**Antibiotic therapy before mNGS results**	**Antibiotic therapy after mNGS results**	**Outcome**
**Patient A**	Pneumonia, Kidney Transplantation Recipient Combined BK Nephropathy	Pulmonary Nocardiosis, Lung Infection of Klebsiella pneumoniae, Kidney Transplantation Recipient Combined BK Nephropathy	Piperacillin tazobactam, → Voriconazole	Ceftriaxone, linezolid → Linezolid, faropenem	Relieved
**Patient B**	Pneumonia, Intracranial mass lesion	Pulmonary Nocardiosis, Cerebral Nocardiosis, Gastrointestinal Hemorrhage	Ceftriaxone	Ceftriaxone, meropenem, sulfamethoxazole → Imipenem cilastatin, ceftriaxone, sulfamethoxazole → Meropenem, amikacin, sulfamethoxazole	Relieved
**Patient C**^a^	Nasopharyngeal carcinoma	NPC, Mixed Pulmonary Infections of Nocardia accompanied by other bacteria	Piperacillin tazobactam → Voriconazole	Amikacin, ceftriaxone, sulfamethoxazole, voriconazole → Amoxicillin and clavulanate potassium, sulfamethoxazole → Sulfamethoxazole	Against-advice discharged
**Patient D**	Pulmonary tuberculosis	Nontuberculosis mycobacteria infection, Pulmonary Nocardiosis	Isonicotinic acid hydrazide, rifampicin, ethambutol, pyrazinamide, clarithromycin	Rifampicin, levofloxacin, sulfamethoxazole, clarithromycin, amikacin	Relieved
**Patient E**	Non-cystic fibrosis bronchiectasis	Pulmonary Nocardiosis	Cefoperazone sulbactam	Sulfamethoxazole → Minocycline^c^	Relieved
**Patient F**	Pulmonary tuberculosis	Pulmonary Nocardiosis, Non-cystic fibrosis bronchiectasis	Ceftriaxone	Ceftriaxone, sulfamethoxazole	Relieved
**Patient G**	Fever origin unknown	Pulmonary Nocardiosis	Ceftriaxone, isonicotinic acid hydrazide, rifampicin, ethambutol, pyrazinamide	Amoxicillin and Clavulanate Potassium, sulfamethoxazole, minocycline	Relieved
**Patient H**^b^	Severe pneumonia	Severe pneumonia, Pulmonary Nocardiosis, Type 1 Respiratory Failure	Piperacillin tazobactam, moxifloxacin	No change	Against-advice discharged
**Patient I**^a^	Hepatoma	Pulmonary Nocardiosis, Hepatoma	-	Ceftriaxone, sulfamethoxazole, amikacin	Relieved
**Patient J**	Pulmonary tuberculosis	Pulmonary Nocardiosis	Ceftriaxone	Sulfamethoxazole	Relieved
**Patient K**	Non-cystic fibrosis bronchiectasis, COPD, SSTI of left foot	Pulmonary Nocardiosis, SSTI of left foot, Non-cystic fibrosis bronchiectasis, COPD	Levofloxacin, fluconazole, cefoperazone sulbactam	Linezolid → Imipenem cilastatin, sulfamethoxazole → Sulfamethoxazole	Relieved
**Patient L**	Non-cystic fibrosis bronchiectasis	Pulmonary Nocardiosis	Piperacillin tazobactam	Sulfamethoxazole	Relieved

*SSTI* Skin soft tissue infection, *COPD* Chronic obstructive pulmonary disease

^a^Lung lesions finding before tumor treatment

^b^Discharge against medical advice before mNGS results

^c^Allergic to Sulfamethoxazole

**Table 6:** Main Diagnosis and treatment of the nND

	**Final main diagnosis**	**Antibiotic therapy before mNGS results**	**Antibiotic therapy after mNGS results**	**Outcome**
**Patient N**	BSI (Escherichia coli), UTI (Enterococcus faecium)	Piperacillin tazobactam → Cefoperazone sulbactam → Imipenem cilastatin → Meropenem → Amikacin, Tigecycline → Ceftazidime → Meropenem	Meropenem, linezolid	Relieved
**Patient M**	Pulmonary aspergillosis	Piperacillin tazobactam	Voriconazole	Relieved
**Patient O**	Pulmonary tuberculosis	Ceftriaxone → Piperacillin tazobactam	Isonicotinic acid hydrazide, rifampicin, ethambutol, pyrazinamide, moxifloxacin	Relieved
**Patient P**^a^	Multiple Myeloma	Valaciclovir → Valaciclovir, piperacillin tazobactam	No change	Relieved
**Patient Q**	Pulmonary tuberculosis	Piperacillin tazobactam	^b^	Against-advice discharged
**Patient R**	Non-cystic fibrosis bronchiectasis	Piperacillin tazobactam	No change	Relieved
**Patient S**	Severe pneumonia^c^, non-small cell lung cancer with metastases	Meropenem, moxifloxacin, Voriconazole, oseltamivir	Meropenem, acyclovir, voriconazole → Piperacillin tazobactam, acyclovir, sulfamethoxazole → Piperacillin tazobactam, caspofungin, sulfamethoxazole → Caspofungin, sulfamethoxazole	Relieved

*BSI* Bloodstream infection, *UTI* Urinary tract infection

^a^Antibiotic trearment used for fever after chemotherapy

^b^Discharge against medical advice before mNGS results

^c^Caused by Pneumocystis jirovecii and cytomegalovirus

## Discussion

Nocardiosis was firstly described by Edmond Nocard in 1888 that could cause granulomatous, abscess as well as pulmonary contention and to date, nocardiosis incidence has been increasing due to growth in patients with immunocompromised status [2]. The diagnosis of nocardial disease commonly depends on the culture methods but there are often difficulties in cultivating the bacteria [4]. Therefore, Nocardiosis constitutes a significant public health care threat due to its under-diagnosis and the lack of sufficient understanding [16]. Recent report indicates that mNGS can be a feasible and practical tool in causative pathogen diagnosis among those difficult cases [11]. Herein, we investigated clinical features of hospitalized patients with *Nocardia* genus detection by mNGS in a tertiary hospital from southern China.

Among the 3756 samples screened, BALF the most common types, which indicated that making a differential diagnosis of pulmonary diseases was usually difficult and clinical physicians from department of ID preferred to performed mNGS in those intractable cases (Fig. 1). As a clinically uncommon pathogen, Nocardiosis can cause a variety of infections involving respiratory system, CNS, cutaneous and soft tissue and others [2]. Similar to others studies conducted in China, pulmonary was the most common attacked site of ND [17, 18]. Excluded CSF and samples from respiratory tract, Nocardiosis detected in others sites was usually not thought to be causative according to physicians (Fig. 2).

Nocardiosis mainly manifest as an opportunistic infection in immunocompromised hosts, especially individuals with altered cellular immunity [19]. However, Nocardiosis can also occur in immunocompetent persons as previously reported and sometimes it can been life-threaten [3, 20–22]. Noteworthy, in the present study, the majority of ND was occurred in the immunocompetent host, especially in the geriatric, reminding that making clinical diagnosis should not neglect the possibility of Nocardiosis among the agedness as well as the normal immunity (Table 1).

Main symptoms of ND can be variable, including fever, cough, dyspnea, which may be present occasionally for months prior to a diagnosis [3, 10, 17]. Asymptomatic nocardiosis was not common and noteworthy, two patients were diagnosis as ND with no clinical presentation during a routine check of chest CT accidentally [4]. Asymptomatic nocardiosis maybe under-diagnosis.

There is a great hard in clinical physicians to differentiate *Nocardia* from fungal or mycobacterial disease as the nonspecific symptoms as well as diverse chest radiography displaying [2, 23]. In our study, T-SPOT can be positive among the ND, which may be erroneously considered as pulmonary tuberculosis. Moreover, chest CT imaging of ND exhibited a variety of displaying, greatly increased the difficult of diagnosis (Table 2). Several laboratory tests results of ND group were obviously different form nND group, but it can not provide feasible evidence to identified ND or nND as it probably due to the complications of patients. Establishment of the nocardiosis diagnosis mainly relied on the time-consuming conventional cultures and isolates identification, and a significant amount of time is needed, which has been reported to be as long as over 6 weeks [24]. Sometimes the culture-dependent methods come out with false results as the growth of nocardiosis can be obscured in a mixed specimen as well as a longer time period may be needed to be incubated for nocardiosis cultures, which might mislead the clinical management strategies. Recent study has illustrated that culture independent methods like mNGS, could not only conduct various *Nocardia* species identification but also greatly cut the detection turnaround time [11, 12, 25]. In our study, most of the patients can establish a definite diagnosis and successfully discharge within 20 days, far shorter than 6 weeks, which might support that mNGS can contribute to the haste of clinical decision making and optimize clinical treatment strategy (Fig. 3).

mNGS is a feasible and practical tool in causative pathogen differentiate diagnosis, such as Patient D, F, J, M, O, Q (Tables 5 and 6). However, our study patients still experienced more than one department in mean as well as at least one kind of antibiotic regimens (exclude Patient I) before the mNGS performed, which greatly increased the health-care costs and the financial burden to families and societies. Maybe mNGS should be tested earlier in those perplexing cases as the difficulties in clarify the diagnosis between ND and nND.

Notably, Patient R was diagnosed as ND nearly after 1 year of the first medical visit as the reads of *Nocardia cyriacigeorgica* greatly increased from 26 (2019–8-16) to 381 (2020–7-1) in BALF. It remind that a followed-up visit as well as a repeated mNGS test of those nND with low reads of *Nocardia* may be necessary.

Most of the study patients received co-sulfonamide treatment and relieved eventually. Sulfonamides were used successfully for many years and has been recommended as the first-line treatment of *Nocardia* infections after solid organ transplantation, but in the patient’s significant renal dysfunction, sulfonamide was desirable to avoid [4].

However, a study indicated that more than half of the *Nocardia* isolates (61% of 765 isolates) were resistance to sulfamethoxazole and nearly half of them were resistance to trimethoprim-sulfamethoxazole (42% of 765 isolates) in USA [26]. Thus, a research recommended that patients with disseminated or severe nocardiosis should accepted initial multi-agent therapy, including co-trimoxazole, amikacin, cephalosporin, carbapenem, minocycline and linezolid [3]. However, two recent researchs indicated those *Nocardia* isolates still presented relatively low resistance rates in co-sulfonamide in China [10, 27].

There are several limitations of our study. Firstly, although conventional cultures are essential as inter- and intraspecies susceptibility patterns of *Nocardia* can vary individually, drug-susceptibility tests were not provided as the technological difficulties of our clinical laboratory [2, 4]. Secondary, the high prices of mNGS may hinder its use in general practice [12] and thus some cases must be missed. Last but not least, the interpretation of mNGS results among clinical physicians maybe inconsistent, which may greatly affect the therapy adjustment.

## Conclusion

In conclusion, our study elucidated on the clinical characteristics of patients with positive *nocardiosis* detected by mNGS. Notably, clinical physicians should focus more on the ND occurred in the immunocompetent host as well as the geriatric. Establishment of ND diagnosis was not easy as the unspecific clinical presentation and sometimes even was asymptomatic presentation. These patients may experience several departments and multiple kinds of antibiotic before an identified diagnosis. Consequently, as a feasible and practical tool in causative pathogen diagnosis, mNGS should be conducted in a early stage. Moreover, repeated mNGS tests maybe necessary among those nND with low reads of *Nocardia* in the initial mNGS test. mNGS tests should become increasingly widespread in the future.

Although there is high resistance of sulfamethoxazole among the *Nocardia* isolates of United States, co-sulfonamide treatment should still be the first choice of ND in China.

### Supplementary Information

**Additional file 1: Supplementary table 1.** Patients characterics of ND.

**Additional file 2: Supplementary table 2.** Patients characterics of nND.

## Data Availability

The datasets generated and analysed during the current study are not publicly available due to privacy or ethical restrictions but are available from the corresponding author on reasonable request.
